# Mitochondrial Genome Study Identifies Association Between Primary Open-Angle Glaucoma and Variants in *MT-CYB, MT-ND4* Genes and Haplogroups

**DOI:** 10.3389/fgene.2021.781189

**Published:** 2021-12-16

**Authors:** Valeria Lo Faro, Ilja M. Nolte, Jacoline B. Ten Brink, Harold Snieder, Nomdo M. Jansonius, Arthur A. Bergen

**Affiliations:** ^1^ Department of Ophthalmology, University of Groningen, University Medical Center Groningen, Groningen, Netherlands; ^2^ Department of Clinical Genetics, Amsterdam University Medical Center (AMC), Amsterdam, Netherlands; ^3^ Department of Epidemiology, University of Groningen, University Medical Center Groningen, Groningen, Netherlands; ^4^ Department of Ophthalmology, Amsterdam UMC, Amsterdam, Netherlands

**Keywords:** mitochondrial polymorphism, mitochondrial haplogroup, primary open-angle glaucoma (POAG), MT-CYB, MT-ND4, haplogroup K, genetic association study

## Abstract

**Background and purpose:** Primary open-angle glaucoma (POAG) is an optic neuropathy characterized by death of retinal ganglion cells and atrophy of the optic nerve head. The susceptibility of the optic nerve to damage has been shown to be mediated by mitochondrial dysfunction. In this study, we aimed to determine a possible association between mitochondrial SNPs or haplogroups and POAG.

**Methods:** Mitochondrial DNA single nucleotide polymorphisms (mtSNPs) were genotyped using the Illumina Infinium Global Screening Array-24 (GSA) 700K array set. Genetic analyses were performed in a POAG case-control study involving the cohorts, Groningen Longitudinal Glaucoma Study-Lifelines Cohort Study and Amsterdam Glaucoma Study, including 721 patients and 1951 controls in total. We excluded samples not passing quality control for nuclear genotypes and samples with low call rate for mitochondrial variation. The mitochondrial variants were analyzed both as SNPs and haplogroups. These were determined with the bioinformatics software HaploGrep, and logistic regression analysis was used for the association, as well as for SNPs.

**Results:** Meta-analysis of the results from both cohorts revealed a significant association between POAG and the allele A of rs2853496 [odds ratio (OR) = 0.64; *p* = 0.006] within the *MT-ND4* gene, and for the T allele of rs35788393 (OR = 0.75; *p* = 0.041) located in the *MT-CYB* gene. In the mitochondrial haplogroup analysis, the most significant *p*-value was reached by haplogroup K (*p* = 1.2 × 10^−05^), which increases the risk of POAG with an OR of 5.8 (95% CI 2.7–13.1).

**Conclusion:** We identified an association between POAG and polymorphisms in the mitochondrial genes *MT-ND4* (rs2853496) and *MT-CYB* (rs35788393), and with haplogroup K. The present study provides further evidence that mitochondrial genome variations are implicated in POAG. Further genetic and functional studies are required to substantiate the association between mitochondrial gene polymorphisms and POAG and to define the pathophysiological mechanisms of mitochondrial dysfunction in glaucoma.

## Introduction

Primary open-angle glaucoma (POAG) is a complex and chronic eye disease characterized by progressive death of retinal ganglion cells (RGCs), which manifests itself initially as visual field loss. Untreated POAG ultimately leads to irreversible blindness (Tham et al., 2014). High intraocular pressure (IOP) is the first and major risk factor identified in patients with POAG (Shaffer, 1996), in addition other known risk factors are advanced age, myopia, ethnicity, and positive family history for POAG (Gramer and Grehn, 2012). Nowadays, the only efficacious therapy for protecting the RCGs (from which the axons form the optic nerve) in POAG is directed towards decreasing IOP. POAG is also defined as a genetically complex disease because many genes have been associated with this condition (Thorleifsson et al., 2010; Burdon et al., 2011; Ramdas et al., 2011; Wiggs et al., 2011; Cao et al., 2012; Osman et al., 2012; Liu et al., 2013; Chen et al., 2014; Gharahkhani et al., 2014; Springelkamp et al., 2015; Trikha et al., 2015; Choquet et al., 2018; Pasquale, 2019). Rearrangements in the DNA and multiple disease genes have been implicated in the pathogenesis of POAG (Davis et al., 2011; Janssen et al., 2013; Liu et al., 2014; Lo Faro et al., 2021). Known disease genes include myocilin (*MYOC*), optineurin (*OPTN*), WD repeat domain 36 (*WDR36*), cytochrome P450 family 1 subfamily B polypeptide 1 (*CYP1B1*), and TANK-binding kinase 1 (*TBK1*) (Janssen et al., 2013). So far, more than 120 chromosomal loci have been discovered through genome-wide association studies (Burdon et al., 2011; Ramdas et al., 2011; Choquet et al., 2018; Gharahkhani et al., 2021). Even so, the disease genes and genetic risk factors identified only explain a small proportion of POAG heritability, and contribute relatively little to understanding the pathogenetic mechanisms. To date, the majority of the genetic studies aim to identify the causes underlying POAG by focusing on the nuclear genome. We and other researchers have postulated that at least part of the remaining POAG heritability might be found in variants in the mitochondrial DNA (mtDNA) (Lascaratos et al., 2012; Osiewacz, 2014; Williams et al., 2017).

The mitochondrion is an organelle mainly involved in the production of cellular energy with distinct extrachromosomal circular and double-stranded molecules of DNA. The mtDNA is transmitted through the maternal germline and it has a unique subcellular transcription and replication machinery (Vincent and Picard et al., 2018). Mutations in the mtDNA have previously been implicated in cellular energy deficits that lead to ocular degenerative disease (Yu-Wai-Man et al., 2011). Indeed, the mitochondria can be considered as a “power plant” for the cell, then it is expected that mitochondrial disorders tend to affect more frequently tissues with high energy demand, such as the retina, brain, muscles, heart, and endocrine systems (Wallace, 2010). A characteristic of the mitochondrial genome (mtGenome) is that it accumulates mutations at a notably faster rate than the nuclear genome. As a result, mtDNA is highly polymorphic. Most likely, this characteristic can be explained by two processes: the lack of protective histones and repair mechanisms, which increase the replication errors, and, given the proximity of mtDNA with the respiratory chain complexes, the exposure to reactive oxygen species (ROS) (Wallace, 2010; Lascaratos et al., 2012).

Many mitochondrial single-nucleotide polymorphisms (mtSNPs) have become fixed in a variety of populations during human evolution (Yu-Wai-Man et al., 2011; Collins et al., 2016). Due to the exclusive maternal inheritance of mtDNA and the fact that the mtGenome does not recombine, mtSNPs are accumulating and co-transmitted through the maternal lineages (Andersen and Balding et al., 2018). This characteristic allows tracing specific, ancestral patterns of human migration that occurred millennia ago, from Africa to other continents. These specific polymorphic SNP-sets accumulating on the mtDNA, allowed researchers to classify human populations into various mtDNA ‘‘haplogroups”. There are a total of nine known haplogroups that identify individuals with European ancestry. These are named H, I, J, K, T, U, V, W, and X, where haplogroup H represents about 40–45% of the total. According to recent studies, specific haplogroups can influence the development of diseases such as POAG, primary angle-closure glaucoma, exfoliation glaucoma, as well as cancer, diabetes, and late-onset neurodegenerative conditions (Herrnstadt et al., 2002; Abu-Amero et al., 2008; van Oven and Kayser, 2009; Abu-Amero et al., 2011a; Abu-Amero et al., 2011b; Urzua-Traslavina and Carlos, 2014). The association between mitochondrial haplogroups and POAG has been investigated in few studies, with conflicting results: Andrews et al. (2006), in a case-control comparison of 140 POAG patients and 75 healthy individuals from England, did not find a difference in the haplogroup distribution between cases and controls (Andrews et al., 2006). In contrast, Collins et al. (2016) found that in African populations the haplogroups L1c2, L1c2b, and L2 were risk factors for POAG (Collins et al., 2016).

An important aspect in the pathophysiology of POAG and similar optic neuropathies is represented by the increased apoptosis of RGCs, and by the functional decay of the trabecular meshwork (TM) (Izzotti et al., 2011; Almasieh et al., 2012). RGCs contain a high number of mitochondria related to their high energy demand. They are especially vulnerable to oxidative damage caused by mitochondrial dysfunction (Sanchez et al., 2016). Apart from POAG, several other optic neuropathies show axonal RGC loss correlated with mitochondrial dysfunction (Howell, 1997; Howell, 2003). Two examples are Leber’s Hereditary Optic Neuropathy (LHON; OMIM 535000) and autosomal dominant optic atrophy (DOA; OMIM 165500). While LHON is caused by three well-known pathogenic mtDNA mutations in the *MT-ND1, MT-ND4*, and *MT-ND6* genes, DOA is caused by pathogenic mutations within the nuclear *OPA1* gene, which codes for a mitochondrial wall membrane protein (Yu-Wai-Man et al., 2009; Wallace, 2010; Yu-Wai-Man et al., 2011).

Next to mitochondrial damage in the RGCs, Izzotti and coworkers investigated potential mitochondrial damage in the TM, a tissue involved in POAG via its influence on IOP. By comparing mtDNA deletions in TM from glaucoma patients and controls by qRT-PCR, the authors observed that oxidative damage arising from mitochondrial failure plays a role in the functional decay of the TM (Izzotti et al., 2011). Another, independent line of evidence for mitochondrial involvement in POAG has come from investigations of glaucoma-prone mice: retinal levels of nicotinamide adenine dinucleotide (NAD) decreased with age, rendering neurons vulnerable to disease-related insults. Interestingly, the administration of a redox metabolite NAD+ and gene therapy of the expression of *Nmnat1*, a key NAD + producing enzyme, had a protective effect. At the highest dose tested, 93% of eyes did not develop glaucoma symptomatology, compared to 50% for the control group (Williams et al., 2017).

Given the hypothesis that RGCs degeneration and functional decay of TM in POAG are influenced by mitochondrial dysfunction, we aimed to explore whether POAG is associated with variations in the mtGenome. To this purpose, we conducted two different association analyses further described below. First, we analyzed mtDNA SNPs in order to detect genetic variations potentially associated with the disease. Second, we analyzed the role of major haplogroups.

## Materials and Methods

### Study Subjects

We performed our association analysis using two case-control studies. The first case-control study consisted of glaucoma cases from the Groningen Longitudinal Glaucoma Study (GLGS), of which a subset of the POAG patients (see below) was genotyped (*n* = 592) (Heeg et al., 2005). The controls (*n* = 1841) were selected from the Lifelines Cohort Study and Biobank (LL) and came from the same geographical region as the GLGS cases. This cohort is addressed in this study as the GLGS-LL cohort. They were age-matched with a 1:3 ratio, using the R package MatchIt with nearest-neighbor matching (Ho et al., 2011). The second case-control study consisted of glaucoma cases (*n* = 129) and controls (*n* = 110) from the Amsterdam Glaucoma Study (AGS) (Ramdas et al., 2011). All the participants were Dutch and of European-ancestry.

The original GLGS cohort has been described in detail by Heeg and colleagues (Heeg et al., 2005). After the inclusion of the initial cohort in 2000–2001, the GLGS continued as a dynamic population, that is, new participants were added during follow-up. We included glaucoma patients who visited the outpatient department of the UMCG in 2015 and who gave written informed consent for a blood sample being taken for genetic analyses. In the GLGS, glaucoma patients showed glaucomatous visual field (VF) loss in at least one eye. For glaucomatous baseline VF loss, two consecutive tests had to be abnormal in at least one eye. Defects had to be compatible with glaucoma and without any other explanation. A VF test before the two baseline tests was discarded to reduce the influence of learning. Those with pseudoexfoliative or pigment dispersion glaucoma or a history of angle-closure or secondary glaucoma were excluded for the current analysis, leaving only POAG patients.

The LL is a multi-disciplinary prospective population-based cohort study examining in a unique three-generation design the health and health-related behaviours of 167,729 persons living in the North of Netherlands. It employs a broad range of investigative procedures in assessing the biomedical, socio-demographic, behavioural, physical and psychological factors which contribute to the health and disease of the general population, with a special focus on multi-morbidity and complex genetics. Participants completed questionnaires, underwent physical examinations, and biological samples including DNA were collected (Scholtens et al., 2015). For the current study, we only included participants without glaucoma and aged 60 years or older. Glaucoma phenotype was defined using a questionnaire-based glaucoma proxy, a classification algorithm built on questions regarding self-reported eye diseases and glaucoma-specific visual complaints (Neustaeter et al., 2020). Participants were classified as having definite, probable, or possible glaucoma, or as healthy. Lifelines controls used in this study were those individuals classified by the proxy as healthy and from whom the genotyping data was available.

The AGS study includes glaucoma cases and healthy controls collected from eye clinics, meetings of the glaucoma patients’ association, nursing homes, and fairs for the elderly from all over the Netherlands. The AGS patients underwent ophthalmoscopy and biomicroscopy with a 90-diopter lens, and digital stereo images of the optic nerve head were taken after mydriatic drops. POAG cases had to have glaucomatous optic neuropathy vertical cup-disc ratio (VCDR) > 0.7 with corresponding glaucomatous visual field loss in at least one eye or a VCDR ≥ 0.8 when no visual field was available (Ramdas et al., 2011). Control subjects from the AGS cohort were selected from unrelated individuals, aged 60 years or older with a VCDR ≤ 0.6 on ophthalmoscopy and fundus photography, and without eye abnormalities.

### Genotyping

Genomic DNA was extracted from the peripheral blood and all individuals were genotyped using the Illumina Infinium Global Screening Array® (GSA) MultiEthnic Disease beadchip version. This array contains approximately 700,000 SNPs and combines multi-ethnic genome-wide content, curated clinical research variants, and quality control (QC) markers. Specifically, for the mtDNA, this array covers 140 mtDNA SNPs. For these latter SNPs, the raw probe intensities were combined in one dataset and called together with Opticall using the -MT option (Shah et al., 2012). To obtain position, strand orientation, and reference allele of mtDNA for our data, we aligned the genotypes with the Cambridge Reference Sequence (rCRS) (ENCODE Project Consortium 2012). For further analysis, we considered only variants that could be mapped perfectly with the reference panel. Next, the following quality control (QC) criteria were applied to SNPs level: 95% call rate per mtDNA SNP in the combined set of case and control individuals and heterozygote mitochondrial genotypes were set to missing, allowing only homozygous calls. At DNA sample level the quality control was first conducted in the autosomal chromosomes and exclusion criteria were 1) duplicated sample, 2) excessive heterozygosity rate, 3) sex discordance, 4) the presence of first and/or second-degree relatives (pi-hat >0.20), and 5) non-European ancestry.

The genotyping datasets of AGS and GLGS-LL were then imputed in IMPUTE2 (Howie et al., 2009). Before the imputation, monomorphic variants were removed and all samples were assigned to male sex to allow haploid imputation. Then we followed instructions for the imputation of chromosome X. The reference panel used contained 36,960 sequences aligned to mtDNA sequences (McInerney et al., 2021). Variants with imputation quality score less than 0.3 and monomorphic variants were excluded.

### Statistical Analysis

To test for association of the mtDNA SNP markers with glaucoma, logistic regressions were conducted separately for GLGS-LL and AGS, with POAG as outcome and SNP as independent variable, adjusting for age and sex. We choose to analyze the two cohorts separately to avoid risk of batch effects and false positive results caused by population stratification. Only SNPs with a minor allele frequency (MAF) > 1% were included in this analysis. To estimate the risks of POAG, odds ratios (ORs) and 95% confidence intervals (CIs) were calculated. Analyses were also performed stratified by sex. The genetic analyses were conducted using PLINK v1.90 (Purcell et al., 2007). Subsequently, we combined the results of the two cohorts by a fixed effects inverse variance weighted meta-analysis using METAL software in which double genomic control was applied (Willer et al., 2010).

A second association analysis was done in the two cohorts separately on reconstructed haplogroups. Since the mtDNA does not recombine, it behaves like a single locus with many alleles making all variants correlated with each other. Mitochondrial haplogroups were estimated from the directly genotyped variants and the haplogroup of each individual was determined with HaploGrep, available at https://haplogrep.i-med.ac.at/ (van Oven and Kayser, 2009; Kloss-Brandstätter et al., 2011). All 140 mtDNA SNPs were entered for the haplogroup determination, and each individual’s haplogroup was determined based on PhyloTree build 17 (implemented in HaploGrep 2.1.21.jar). We only included haplogroup assignments with a quality score above 80% (determined by HaploGrep), and with a frequency above 1% (van Oven and Kayser, 2009; Kloss-Brandstätter et al., 2011). For the association test, sub-haplogroups were first assigned to their respective major haplogroups. We tested each haplogroup against haplogroup H, which is the most common European haplogroup (22.9% in our dataset; see Discussion section), as the reference using logistic regression, adjusting for age and sex (Torroni and Wallace et al., 1994). Chi-square analysis was conducted to determine the effect of specified sub-haplogroup K (K1, K1a1, K1a11, K1a1b2a1, K1a24a, K1a4a, K1a4a1a2a, K1b2a, and K1c1).

Given the hypothesis-free approach of our exploratory study and the risk to test not independent SNPs due to high linkage disequilibrium in mtDNA, we reported nominal significant *p*-values (≤0.05) (Andersen and Balding et al., 2018). The analyses were performed using RStudio.

### Gene Expression

In order to evaluate gene expression of significant SNPs, we queried the EyeIntegration database v1.05 (https://eyeintegration.nei.nih.gov/) in cornea, retina, and retinal pigment epithelium (RPE) (Bryan et al., 2018). This database is created by investigators at the National Eye Institute (National Institutes of Health) and contains publicly deposited RNA-seq datasets from human ocular tissues (Bryan et al., 2018). Gene correlation networks were constructed using the kWithin metric to measure the connectivity. Genes with higher connectivity are, theoretically, more likely to be important in gene regulation as perturbations in them will affect the system more than less connected genes. Identified genes, either those closest to significant SNPs or resulting from the gene correlation network, were queried in the Online Mendelian Inheritance in Man (OMIM) database, to identify associated phenotypes (Hamosh, 2004).

### Ethics Statement

The study followed the tenets of the Declaration of Helsinki and the ethics board of the University Medical Center of Amsterdam (UMC) approved this research (METc submission # 2013_327). All participants provided written informed consent.

## Results

A total of 2,672 individuals were included. Table 1 shows the demographics of both cohorts.

**TABLE 1:** Characteristics of the GLGS-LL and AGS cohorts.

	GLGS-LL		AGS	
	Cases (*n* = 592)	Controls (*n* = 1841)	Cases (*n* = 129)	Controls (*n* = 110)
Age [median (IQR)]	73 (66, 80)	70 (68, 73)	73 (65, 79)	72 (68, 79)
Sex, female, n (%)	264 (44.5)	801 (43.5)	57 (44.1)	67 (60.9)

IQR: interquartile range.

### Single SNP Analysis

In total, 140 mtSNPs were initially screened on the DNA of our case control populations. Forty SNPs in the GLGS-LL dataset and 39 SNPs in the AGS cohort passed the quality control criteria previously mentioned (see Subjects and Methods section) and were used for further analysis. In the GLGS-LL cohort we excluded 51 SNPs that were monomorphic or had a low MAF. We excluded 49 additional ones for a relatively high missing genotype rate. In the AGS cohort, 101 mtDNA SNPs were monomorphic or had a low MAF but we did not exclude any SNP for high missing genotype rate. After imputation, 69 and 63 variants were retained in the GLGS-LL and AGS cohorts, respectively.

Logistic regression analyses were conducted separately in the GLGS-LL and AGS datasets, where POAG was modelled as the dependent variable and the genotyped variants as the independent variable (Table 2).

**TABLE 2:** Significant mtDNA single-nucleotide polymorphisms associated with POAG in the GLGS-LL and AGS cohorts, separated for sex.

	Marker	Single-nucleotide polymorphism	Nearest gene	Effect allele	No effect allele	Frq* Case/Control	Odds ratio (95% CI**)	*p*-value
GLGS-LL								
Both sex	*MitoG11915A*	rs2853496	*MT-ND4*	A	G	0.006/ 0.023	0.27 (0.01–0.78)	0.015
Female	*MitoG11915A*	rs2853496	*MT-ND4*	A	G	0.003/0.03	0.12 (0.016–0.88)	0.038
Male	*2010–08-MT-723*	rs879162984	D-loop	A	G	0.036/0.015	2.41 (1.12–5.2)	0.024
	*MitoA16163G*	rs41466049	*MT-CYB*	G	A	0.073/0.046	1.77 (1.06–2.96)	0.027
AGS								
Both sex	*rs2853826*	*rs2853826*	*MT-ND3*	G	A	0.24/0.10	2.29 (1.11–4.69)	0.023
	*MitoT12706C*	rs193302956	*MT-ND5*	T	C	0.11/0.045	3.53 (1.12–11.18)	0.031
	*MitoT491C*	rs28625645	D-loop	C	T	0.10/ 0.03	3.21 (1.001–10.34)	0.049
Female	*rs2853826*	*rs2853826*	*MT-ND3*	G	A	0.22/0.075	3.69 (1.22–11.17)	0.02

Abbreviations: *Frq, frequency of effect allele; **CI, confidence interval.

These results were then meta-analyzed for 32 SNPs in common between the two datasets, revealing nominal associations in the mtDNA SNPs rs2853496 (*p* = 0.006) and rs35788393 (*p* = 0.041) with POAG, in the sex combined cohorts (Table 3).

**TABLE 3:** Nominal significant mtDNA SNPs associated with POAG from the meta-analysis of the GLGS-LL and AGS cohorts, conducted in both sexes.

SNP	Position	Gene	Effect allele	No effect allele	Odds ratio	*p*-value	Hetisq	Hetpval
rs2853496	11,914	*MT-ND4*	A	G	0.64	0.006	0	0.92
rs35788393	15,904	*MT-CYB*	T	C	0.75	0.041	0	0.93

SNP—single nucleotide polymorphism; Hetpval—heterogeneity *p*-values from Cochrane’s Q statistic; Hetisq—heterogeneity index (0–100%).

### Haplogroup Analysis

In addition to single SNP association analyses, we also conducted an association test between mitochondrial haplogroups and POAG. The inclusion threshold for this analysis (see methods section) was reached by a total of 235 individuals, i.e., 143 cases and 92 controls from the two cohorts that fulfilled the quality criteria. The identified haplogroups were all assigned to one of the European major haplogroups (H, K, and U). Table 4 summarizes haplogroup frequency distributions for the cases and controls.

**TABLE 4:** Haplogroup frequency distributions and their association with the risk of POAG.

Haplogroup	Frq* females N (%)	Frq* males N (%)	Frq* cases N (%)	Frq* controls N (%)	Odds ratio	95% CI**	*p*-value
H	31 (13.2)	23 (9.7)	20 (8.6)	34 (14.5)	Reference	Reference	Reference
K	29 (12.3)	45 (19.2)	58 (24.6)	16 (6.8)	5.8	2.7–13.1	1.2 × 10-05
U	62 (26.4)	45 (19.2)	65 (27.7)	42 (17.8)	2.61	1.3–5.2	0.005

Abbreviations: *Frq, frequency of effect allele; **CI, confidence interval.

The haplogroup K showed an increased risk effect for POAG, with an OR = 5.8 (95% CI = 2.7–13.1; *p* = 1.2 × 10^−05^) and the haplogroup U an OR = 2.6 (95% CI = 1.3–5.2; *p* = 0.005). We further observed that the haplogroup U was the most common haplogroup (45.5%), followed by haplogroups K (31.4%) and H (22.9%). None of these K sub-haplogroups showed any statistical significance (data not shown). The haplogroup U is phylogenetically connected with the haplogroup K, and was also significantly associated with increased risk of POAG. Therefore, we repeated our analysis combining the haplogroups U and K in the UK cluster and found that UK frequency also differed significantly from the control group (odds ratio 3.5, 95% CI = 1.8–6.7; *p* = 0.00012).

### Gene Expression

**FIGURE 1:**
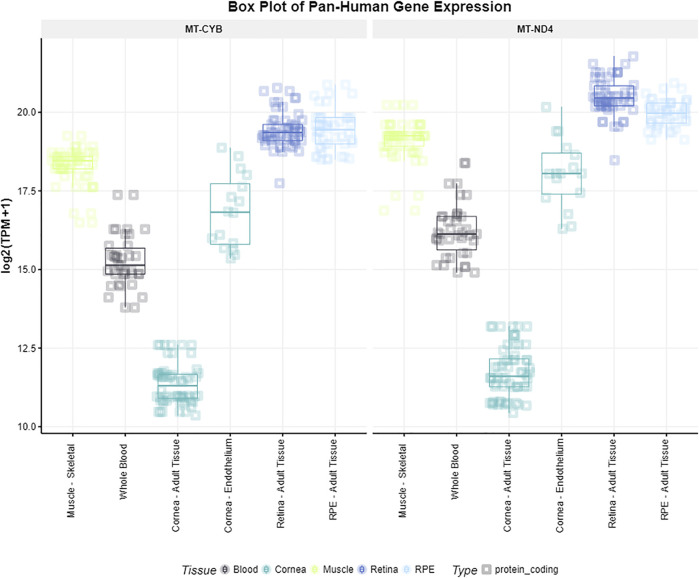
Gene expression of *MT-CYB* and *MT-ND4* in different tissues. Gene expression levels of the genes *MT-CYB* and *MT-ND4* according to the EyeIntegration database v1.05 for muscle skeletal, whole blood, cornea, retina and RPE tissues.

We queried the two genes located closest to the two identified SNPs (*MT-CYB* and *MT-ND4*) for expression in ocular and nonocular tissues in the EyeIntegration database. We found the highest expression of both *MT-CYB* and *MT-ND4* in the following tissues, respectively: adult retina of 19.56 log2 (TPM + 1) and 20.60 log2 (TPM + 1), and RPE of 19.59 log2 (TPM + 1) and 20.03 log2 (TPM + 1). The lowest gene expression was reported in the cornea [11.50 log2 (TPM + 1) and 11.97 log2 (TPM + 1)] (Figure 1).

Gene expression levels of the genes *MT-CYB* and *MT-ND4* according to the EyeIntegration database v1.05 for muscle skeletal, whole blood, cornea, retina and RPE tissues.

We also queried in the EyeIntegration database v1.05 the retina network to examine which genes were the most connected in this network. In the retina network, *MT-CYB* and *MT-ND4* genes have high connectivity with the *POMGNT1* gene (kWithin = 18.447). Genes with higher connectivity are, theoretically, more likely to be important in gene regulation because perturbations in them will affect more the system compared to the effect in less connected genes. When we queried the *POMGNT1* gene in the OMIM database, we identified eye phenotypes linked to muscular dystrophy-dystroglycanopathy, in which patients have congenital glaucoma and retinitis pigmentosa (Parton, 2003).

## Discussion

In this study, we investigated the possible involvement of genetic variation in mitochondria in POAG, by performing an association analysis for mitochondrial SNPs and haplogroups in 721 patients with POAG and 1951 healthy individuals. Based on evidence derived from combined analysis of our datasets, we concluded that two mtSNPs (rs2853496 and rs35788393) are nominal associated with POAG. Our data suggest that the A allele of rs2853496, within the *MT-ND4* gene, and the T allele of rs35788393, located in the *MT-CYB* gene, have a protective effect. With respect to mitochondrial haplogroups, our analyses identified haplogroup K as highly associated with an increased risk of POAG (OR = 5.8; 95% CI = 2.7–13.1; *p* = 1.2 × 10^−5^).

Our findings are consistent with evidence from the literature that suggest a potential role of the mtGenome, and more specifically of the genes *MT-ND4* and *MTCYB* in optic neuropathies or glaucoma (Cortopassi and Arnheim et al., 1990; Votruba et al., 2004; Abu-Amero et al., 2006). The *MT-ND4* gene is a protein-coding gene located in the mtDNA, encoding for subunit 4 of complex I (NADH ubiquinone oxidoreductase) (MT-ND4, 2021). The complex I is the first enzyme of the respiratory chain, a vulnerable site to oxidative stress, also involved in cellular functions like apoptosis (Ferguson et al., 1976). SNPs in subunit 4 of *MT-ND4* can affect the first step of the electron transport chain. Therefore, these mutations may have an impact on mitochondrial respiratory chain function and could result in an alteration of the cellular energy metabolism.

Genetic variations in the *MT-ND4* are implicated in other optic neuropathies. This is the case of LHON, where one of the most prevalent variants that accounts for more than 70% of all cases is the m.11778G > A, located in the *MT-ND4* gene (Yu-Wai-Man et al., 2014; Mancuso and Klopstock et al., 2019). LHON is one of the most common inherited optic neuropathies and it is characterized by bilateral optic atrophy and loss of central vision due to loss of RGCs (Sadun, 2002; Yu-Wai-Man et al., 2011). MtDNA mutations associated with LHON have also been described in animal models: mice with a mutation in the *mt-Nd4* gene show nerve atrophy and RGCs degeneration. Both conditions are also characteristics of LHON in humans (Divi et al., 2007; Koilkonda and Guy, 2011). In contrast to the mitochondrial mutations identified in LHON cases, in the mitochondrial genome of POAG patients the majority of the mutations were somatic transversions (a replacement of a purine with a pyrimidine, or vice versa), caused by the accumulation of oxidative stress over time (Abu-Amero et al., 2006).

In this study, we also report an association between POAG and the mtDNA variation rs35788393 in the *MT-CYB* gene. The protein product of this gene is involved in the oxidative phosphorylation system. This system is composed of five complexes where the *MT-CYB* gene encodes for the cytochrome b of complex III (that catalyzes the transfer of electrons from ubiquinol to cytochrome c), the only one solely encoded by a mitochondrial gene (Chaussenot et al., 2018). Pathogenic mutations in the *MT-CYB* gene can disrupt the normal activity of the electron transport chain and affect the production of ATP by increasing the production of ROS. This leads to damage of cellular proteins, lipids and nucleic acids via oxidation reactions (West et al., 2011). So far, mutations in *MT-CYB* have been associated with LHON, retinitis pigmentosa and cataract (Brown et al., 1992; Wibrand et al., 2001; Schuelke et al., 2002; Ronchi et al., 2011). Since the *MT-CYB* gene is involved in the production of ATP in the electron transport chain, it is pivotal to explore the possible role of this gene in POAG in future studies.

Genetic variations in both *MT-CYB* and *MT-ND4* genes are able to destabilize the so-called mitochondrial super complex: the physical interaction between mitochondrial complex-I and complex-III. The destabilization of this complex leads to the loss of complex I activity, the major entry point for electrons to the respiratory chain (Hudson et al., 2007). In transgenic mice, loss of complex-I activity showed an increase of ROS levels in the RCGs and optic nerve degeneration (Qi et al., 2003). In human, glaucomatous TM cells have been reported to be more sensitive to the inhibition of complex-I (Banerjee et al., 2013). Indeed, damages in complex-I were observed to contribute to the progressive loss of TM cells in POAG patients due to the excessive mitochondrial ROS production and to the attenuation of the mitochondrial membrane. This decrease reduces the ATP synthesis, driving the cells towards apoptosis (He et al., 2008). Another study comparing both POAG and LHON lymphoblasts found an impairment of the complex-I, where functional defects of this complex were milder in POAG than LHON. This is in accordance with the less severe development of the disease in POAG (Van Bergen et al., 2015). However, more comprehensive investigations are still necessary to define the regulatory function of complex-I that in turn might lead to the increase of the oxidative stress and favor the glaucomatous condition.

To add biological context to our study, we also evaluated bioinformatically which genes were the most highly connected with *MT-CYB* and *MT-ND4* in the retina network (Bryan et al., 2018). Genes highly connected indicate that they are more likely to have an effect in gene regulation. By querying the retina network, we implicated the *POMGNT1* gene (protein O-mannose beta-1,2-N-acetylglucosaminyltransferase-1). *POMGNT1* synthesizes a unique O-mannose sugar chain on α-dystroglycan, the extracellular protein that binds laminin α2 in the extracellular matrix. Reported mutations in the *POMGNT1* gene have enlightened its role in four genetic muscular dystrophy disease entities: 1) Walker–Warburg syndrome (OMIM #253280), 2) the muscle-eye-brain disease (OMIM #253280)—for which patients show ocular symptoms as retinal degeneration, optic atrophy and congenital glaucoma—3) congenital muscular dystrophy with mental retardation (OMIM #613151), and 4) retinitis pigmentosa (OMIM #606822) (Pihko et al., 1995; Yoshida et al., 2001; Godfrey et al., 2007; Clement et al., 2008; Wang et al., 2016; Xu et al., 2016). The dystroglycan gene (*DAG1*) encodes for α-dystroglycan and β-dystroglycan (Holt et al., 2000). In the retina, dystroglycan is highly expressed in Müller glial, rods and cones at the outer plexiform layer, and plays an important role in retinal function and survival (Schmitz et al., 1993; Montanaro et al., 1995; Blank et al., 1999; Jastrow et al., 2006). The *DAG1* gene is also linked to Muscular dystrophy-dystroglycanopathy (congenital with brain and eye anomalies, OMIM #616538). Using zebrafish animal models, Gupta et al. demonstrated that dystroglycan deficiency caused abnormal development of ganglion cells, lens, and cornea (Gupta et al., 2011). In addition, in a consanguineous Israeli-Arab family a homozygous loss-of-function mutation in the *DAG1* gene was detected in infants with a congenital phenotype consistent with Walker–Warburg syndrome. Ocular features in those infants included bilateral corneal opacity and glaucoma (Riemersma et al., 2015). Taken together, these findings suggest that dystroglycan deficiency is strongly correlated with eye abnormalities, including glaucoma. In our view, further studies on the role of *DAG1* and *POMGNT1* genes in the pathomechanisms underlying POAG are warranted.

Another part of our current investigation in POAG was focused on the analysis of potentially associated mtDNA haplogroups. In order to interpret our results, it is important to consider the variations reported in the population distributions of mitochondrial haplotypes in comparison with the frequencies identified in our study (Torroni and Wallace, 1994). A study conducted in the Netherlands in 680 individuals randomly selected identified that the most common haplogroup was H (45.3%), followed by haplogroups U (25.6%), T (11.6%), J (10.7%) and K (6.3%) (Chaitanya et al., 2016). These frequencies, when present in our POAG dataset, differed from those reported in the general population. Regarding the haplogroup H, it is important to point out that it shows a complex variation with many sub-lineages. In our study, we used data generated by a SNP-chip array, which is able to detect sites that are polymorphic in populations. Therefore, the differences in frequency reported here for the haplogroup H can be attributed to the absence of sites that allows an accurate classification (Loogväli et al., 2004; Pereira et al., 2005). In our study, haplogroup K was the most significant association with POAG. Considering that haplogroup K occurs approximately in 8% of European and 6% in Dutch individuals, we reported a higher frequency of this haplogroup in our POAG cases (24.6%) compared to controls (6.8%). In line with our findings, a meta-analysis conducted in 3,613 individuals affected by LHON from 159 European pedigrees indicated that the risk of visual loss was higher in carriers of the mitochondrial haplogroup K: individual carriers of haplogroup K were more exposed to experience visual loss, whereas individuals carriers of haplogroup H had a lower risk of visual loss (Hudson et al., 2007). The haplogroup association in our study showed a similar outcome: individuals with haplogroup K had a higher risk to develop POAG compared to individuals belonging to haplogroup H. We also observed that the UK cluster shows the same direction of risk. Interestingly, studies conducted in cybrids showed that haplogroup UK are associated with less levels of mitochondrial protein synthesis and respiratory complex IV activities than cybrids from haplogroup H (Gómez-Durán et al., 2010). Therefore, also supported by previous studies, we speculate on the possible link of the mtGenome in the pathogenesis of POAG (Abu-Amero et al., 2011b; Bosley et al., 2011; Collins et al., 2016; Singh et al., 2018). In contrast, a few studies reported that mitochondrial haplogroups did not contribute to the pathogenesis of POAG. Negative associations were found in POAG cohorts from the north east of England, Saudi Arabia and Ghana (Andrews et al., 2006; Abu-Amero et al., 2008, 2012). Combining our data with those of the literature, there is evidence that mitochondrial haplotypes K plays a role in the pathogenesis of POAG, at least in some populations.

### Strengths and Limitations

Our study had several strengths and limitations. Strong points are: the AGS and GLGS datasets are well defined in terms of diagnosis, design, and method of investigation. In fact, these datasets are clinical cohorts, in which POAG patients received diagnoses by experienced physicians, following strict criteria. In addition, the cohorts used in this study were genotyped on the same array and have been processed applying the same quality control procedures. There are also a number of limitations: first, by nature of the study, we focused only on homoplasmic mtDNA variations, detected in blood but not in potentially relevant ocular tissues. Furthermore, we cannot exclude potential additional influence of nuclear DNA of mitochondrial origin, other genetic variations elsewhere or non-genetic factors. Second, we performed our analyses in samples of European ancestry and for this reason our findings are not, without more research, transferable to other populations. Third, replications in other populations of our results are necessary to corroborate their association with POAG.

## Conclusion

In our study, we identified associations between mitochondrial polymorphisms in the *MT-ND4* and *MT-CYB* genes and POAG, and reported that individuals carrying mtDNA haplogroup K were at the highest risk of developing this eye disease. Our findings support the hypothesis that mitochondria have a role in the pathogenesis of POAG. Nonetheless, further genetic and functional studies are still required to highlight the role of mitochondrial genes, in relation to POAG pathophysiology.

## Lifelines Cohort Study

**Raul Aguirre-Gamboa**, Department of Genetics, University of Groningen, University Medical Center Groningen, Netherlands; **Patrick Deelen**, Department of Genetics, University of Groningen, University Medical Center Groningen, Netherlands; **Lude Franke**, Department of Genetics, University of Groningen, University Medical Center Groningen, Netherlands; **Jan A. Kuivenhoven**, Department of Pediatrics, University of Groningen, University Medical Center Groningen, Netherlands; **Esteban A. Lopera Maya**, Department of Genetics, University of Groningen, University Medical Center Groningen, Netherlands; **Ilja M. Nolte**, Department of Epidemiology, University of Groningen, University Medical Center Groningen, Netherlands; **Serena Sanna**, Department of Pediatrics, University of Groningen, University Medical Center Groningen, Netherlands; **Harold Snieder**, Department of Epidemiology, University of Groningen, University Medical Center Groningen, Netherlands; **Morris A. Swertz**, Department of Genetics, University of Groningen, University Medical Center Groningen, Netherlands; **Peter M. Visscher**, Department of Epidemiology, University of Groningen, University Medical Center Groningen, Netherlands, and Institute for Molecular Bioscience, The University of Queensland, Brisbane, Queensland, Australia; **Judith M Vonk**, Department of Epidemiology, University of Groningen, University Medical Center Groningen, Netherlands; **Cisca Wijmenga**, Department of Genetics, University of Groningen, University Medical Center Groningen, Netherlands.

## Data Availability

The data that support the findings of this study are available from Lifelines Biobank but restrictions apply to the availability of these data, which were used under license for the current study, and so are not publicly available. Data are however available from the authors upon reasonable request and with permission of Lifelines.
